# A Cross-Sectional Study on the Prevalence of Asthma-Chronic Obstructive Pulmonary Disease Overlap in Patients Attending the Pulmonology Outpatient Department at Apollo Institute of Medical Sciences and Research, Chittoor, India

**DOI:** 10.7759/cureus.118425

**Published:** 2026-10-22

**Authors:** C V Nandeshwara Reddy, Saikrishna P, Dimple Swetha Mopati, Cheedella V H N Sandhya, P Sree Ramulu

**Affiliations:** 1 Respiratory Medicine, Apollo Institute of Medical Sciences and Research, Chittoor, IND

**Keywords:** aco: asthma-copd overlap, bronchial asthma copd, fev1/fvc ratio, prevalence, sex differences

## Abstract

Background

Bronchial asthma and chronic obstructive pulmonary disease (COPD) exhibit distinct clinical characteristics; nevertheless, it is common to come across patients who display clinical signs of both conditions in clinical settings. The identification of this phenomenon has resulted in the adoption of the term "asthma-COPD overlap" (ACO), which characterizes a set of clinical characteristics rather than defining a singular entity. Different studies have indicated a prevalence of 15 to 55% for ACO. Individuals diagnosed with ACO experience more frequent severe episodes, leading to higher rate of inpatient care, emergency visits, and increased healthcare expenses compared to patients diagnosed with asthma and COPD. The present study was undertaken to determine the prevalence and phenotype of asthma-COPD overlap in patients attending the pulmonology outpatient department at Apollo Institute of Medical Sciences and Research (AIMSR) and District Headquarters Hospital (DHH) in Chittoor, India.

Methods

This was a prospective cross-sectional study conducted in patients attending the pulmonology department at AIMSR and DHH during May 2025 to August 2026. The demographic details were collected from the patients included in the study. All patients underwent Chest X-ray and pulmonary function test. The observations were tabulated and represented in frequency and percentage. The comparisons based on sex were done and the association was assessed using the Chi-square test and Student's t-test.

Results

This study included 79 subjects, of which 35 (44%) were females and 44 (56%) were males. Among females, 18 (51.43%) did not have a prior COPD diagnosis, while 17 (48.57%) were diagnosed with COPD. Among males, 14 (31.82%) did not have a prior COPD diagnosis, whereas 30 (68.18%) were diagnosed with COPD. The mean forced expiratory volume in one second to forced vital capacity (FEV1/FVC) ratio was 0.68±0.19 in female subjects and 0.64±0.17 in males. Among previously diagnosed asthmatics, 12 males and 20 females exhibited ACO, while it was seen in 30 males and 17 females in previously diagnosed COPD patients.

Conclusion

In the present study, the prevalence of ACO based on Global Initiative for Asthma 2016 guidelines in asthmatics was found to be seven in men and 12 in females, but in individuals with COPD, the prevalence was found to be eight in males and three in females.

## Introduction

Asthma and chronic obstructive pulmonary disease (COPD) are the prevailing obstructive lung illnesses encountered in clinical practice. They have common attributes and exhibit comparable clinical symptoms. Furthermore, they typically exhibit similar characteristics when assessed using pulmonary function testing (PFT). Hence, the term "asthma-COPD overlap" (ACO) was employed to characterize instances that exhibit characteristics of both asthma and COPD. The concept of ACO was initially introduced by Gibson in 2009 [1]. 

ACO is defined as an obstructive lung condition with clinical and inflammatory characteristics of asthma and COPD or predominantly COPD combined with bronchodilator responsiveness and elevated peripheral eosinophil count. Despite the increasing description of the condition, the previous Global Initiative for Chronic Obstructive Lung Disease (GOLD) did not recognize ACO as a separate condition due to the lack of a globally accepted definition [2].

The frequently used definition of ACO is the occurrence of airflow obstruction in patients who have a history of asthma and are above the age of 40. As per the Global Initiative for Asthma (GINA) and GOLD, patients typically experience persistent symptoms of chronic airway disease, including cough, wheezing, frequent lower respiratory tract infections, and characteristics of both asthma and COPD [2,3].

Asthma and COPD are respiratory conditions that have distinct clinical characteristics, yet they share a common feature of restrictive or obstructive airflow limitations. Patients who have both COPD and asthma may potentially be diagnosed with ACO, a condition in which the clinical and functional characteristics of both diseases coincide. In 2000, ACO was incorporated into a range of national and international recommendations [4].

Patients with asthma who have a persistent airflow limitation (post-bronchodilator forced expiratory volume in 1 second (FEV1)/forced vital capacity (FVC) <70%) and a smoking history, or patients with COPD who have a history of childhood asthma, eosinophilia, and reversibility in pulmonary function tests (PFTs), should consider ACO as a potential diagnosis. Different studies have indicated various rates of 15%-55% for the prevalence of cases with ACO. Individuals diagnosed with ACO experience more frequent and severe episodes, leading to a higher rate of hospitalizations, emergency visits, and increased healthcare expenses compared to patients diagnosed with asthma and COPD [5].

The present study was undertaken to determine the prevalence and phenotype of ACO in patients attending the pulmonology outpatient department at Apollo Institute of Medical Sciences and Research (AIMSR) and District Headquarters Hospital (DHH), Chittoor. The secondary objective of the study is to assess the airway behaviour of the ACO patient using spirometry.

## Materials and methods

This prospective cross-sectional study included patients diagnosed with COPD and asthma. This study was conducted at AIMSR and DHH, Chittoor, Andhra Pradesh, India, from May 2025 to August 2026 after obtaining institutional ethics approval (PG/029/IEC/AIMSR/2022). The study population was selected based on the calculated sample size (n=Z^2^pq/d^2^), based on the 14.6% prevalence of ACO reported by Daniel et al. from Christian Medical College, Vellore, Tamil Nadu [6]. The calculated sample size at 95% confidence interval and a precision of 8% was 74. Non-probability sampling technique was used for selecting the patients into the study.

The patient data were obtained after taking informed consent. Male and female subjects over 40 years of age, including elderly asthmatics with irreversible airway obstruction on pulmonary function testing (spirometry), asthmatics with fixed airway obstruction, asthmatics with a history of smoking and biomass exposure, and patients with COPD and a history of allergy (to indoor allergens such as animal dander, dust mite droppings, cockroach droppings, and molds, or outdoor allergens such as pollen and molds) were included in the study. Patients with other causes of eosinophilia (such as peripheral eosinophilia or parasitic infections), patients with a positive sputum smear for acid-fast bacilli (AFB), a history of tuberculosis, old tuberculosis, interstitial lung disease, or other comorbidities, and patients with COVID-19 infection, acute exacerbations, contraindications to spirometry, or those unwilling to participate were excluded from the study.

The primary outcome of the study was measure of ACO prevalence based on the GINA guideline 2016 and the secondary outcome was to view airway behavior in ACO subjects [3].

Demographic data were collected from the subjects included in the study. All subjects underwent a chest X-ray that was physician-reported, venipuncture for a complete blood count assessed at a nationally accredited laboratory, and pulmonary function testing (FVC, FEV1, FEV1/FVC, and small airway parameters) via spirometry following European Respiratory Society/American Thoracic Society (ERS/ATS) 2019 standards using a Spirowin device (Spirowin version 2.01a, Genesis Medical Systems Pvt. Ltd., Hyderabad, India). FEV1/FVC ratio <70% of baseline post-bronchodilator was considered obstructive airflow while FEV1/FVC ratio >70% with FVC <80% of predicted value was considered restrictive airflow. Increase in FEV1 by 12% post-bronchodilator administration was considered reversibility [6,7].

Statistical method

Data were entered into Microsoft Excel (Redmond, WA, USA) and analysis was done using SPSS Statistics version 21 (IBM Corp., Armonk, NY, USA). Descriptive and frequency analysis was performed. Frequency, mean and standard deviation were used for representation. Chi-square test and Student’s t-test were performed to obtain the level of significance (P), and a P value <0.05 was considered significant.

## Results

This study included 79 subjects. The study subjects’ characteristics were tabulated in Table 1. Among the study subject’s male preponderance was observed. The age group distribution among both genders was comparable (P=0.46). There was no gender specific association with occupation among the study subjects. Smoking was significantly (P=0.0001) seen in male subjects, while biomass exposure was significantly higher (P=0.007) in female subjects (Table 1).

**Table 1:** Characteristics of study population. Total number of subjects (N)=79; COPD: chronic obstructive pulmonary disease. P value was obtained using Chi-square test. X2 is chi-square value. Reversibility of Asthma and Reversibility of COPD were determined using post-bronchodilator forced expiratory volume in one second (FEV1) or forced vital capacity (FVC) values.

Patient Characteristics		Female N (%)	Male N (%)	Total N (%)	P value	X^2^
Sex		35(44)	44(56)	79(100)	---	---
Age group	45 - 55 year	20(57.14)	20(45.45)	40(50.63)	0.86	2.54
	55 - 65 year	10(28.57)	20(45.45)	30(37.97)		
	65 - 75 year	3(8.57)	2(4.55)	5(6.33)		
	> 75 year	2(5.71)	2(4.55)	4(5.06)		
	Total	35(100)	44(100)	79(100)		
Occupation	Farmer	16(45.71)	24(54.55)	40(50.63)	0.96	0.62
	Office Worker	8(22.86)	8(18.18)	16(20.25)		
	Other Labourer	11(31.43)	12(27.27)	23(29.11)		
	Total	35(100)	44(100)	79(100)		
Smoking Status	Non-Smoker	35(100)	4(9.09)	39(49.37)	0.0001	64.45
	Smoker	0(0)	40(90.91)	40(50.63)		
	Total	35(100)	44(100)	79(100)		
Previously Diagnosed with Asthma	Absent	15(42.86)	32(72.73)	47(59.49)	0.007	7.2
	Present	20(57.14)	12(27.27)	32(40.51)		
	Total	35(100)	44(100)	79(100)		
Biomass Exposure for more than 15 years	Absent	2(5.71)	13(29.55)	15(18.99)	0.007	7.19
	Present	33 (94.29)	31(70.45)	64(81.01)		
	Total	35(100)	44(100)	79(100)		
Previously Diagnosed with COPD	Absent	18(51.43)	14(31.82)	32(40.51)	0.07	3.11
	Present	17(48.57)	30(68.18)	47(59.49)		
	Total	35(100)	44(100)	79(100)		
Reversibility of Asthma	Irreversible	12(60)	7(58.33)	19(24.05)	0.92	0.008
	Reversible	8(40)	5(41.67)	13(16.46)		
	Total	20(100)	12(100)	32(40.51)		
Reversibility of COPD	Irreversible	14(82.35)	22(73.33)	36(45.57)	0.48	0.49
	Reversible	3(17.65)	8(26.67)	11(13.92)		
	Total	17(100)	30(100)	47(59.49)		
Absolute Eosinophilic Count	< 100 cells/μ L	6(17.14)	8(18.18)	14(17.72)	0.55	1.19
	100 - 200 cells/μ L	16(45.71)	15(34.09)	31(39.24)		
	> 300 cells/μ L	13(37.14)	21(47.73)	34(43.04)		
	Total	35(100)	44(100)	79(100)		
Interpretation of Chest X Ray	COPD Changes	17(48.57)	30(68.18)	47(59.49)	0.07	3.11
	Normal	18(51.43)	14(31.82)	32(40.51)		
	Total	35(100)	44(100)	79(100)		

The mean and standard deviation of the FEV1/FVC ratio among patients, categorized by sex within the study population, are depicted in Figure 1. For females, the mean FEV1/FVC ratio was 0.68 with a standard deviation of 0.19. For males, the mean FEV1/FVC ratio was 0.64 with a standard deviation of 0.17. The mean ratios were comparable (P=0.33) between the two genders.

**Figure 1:**
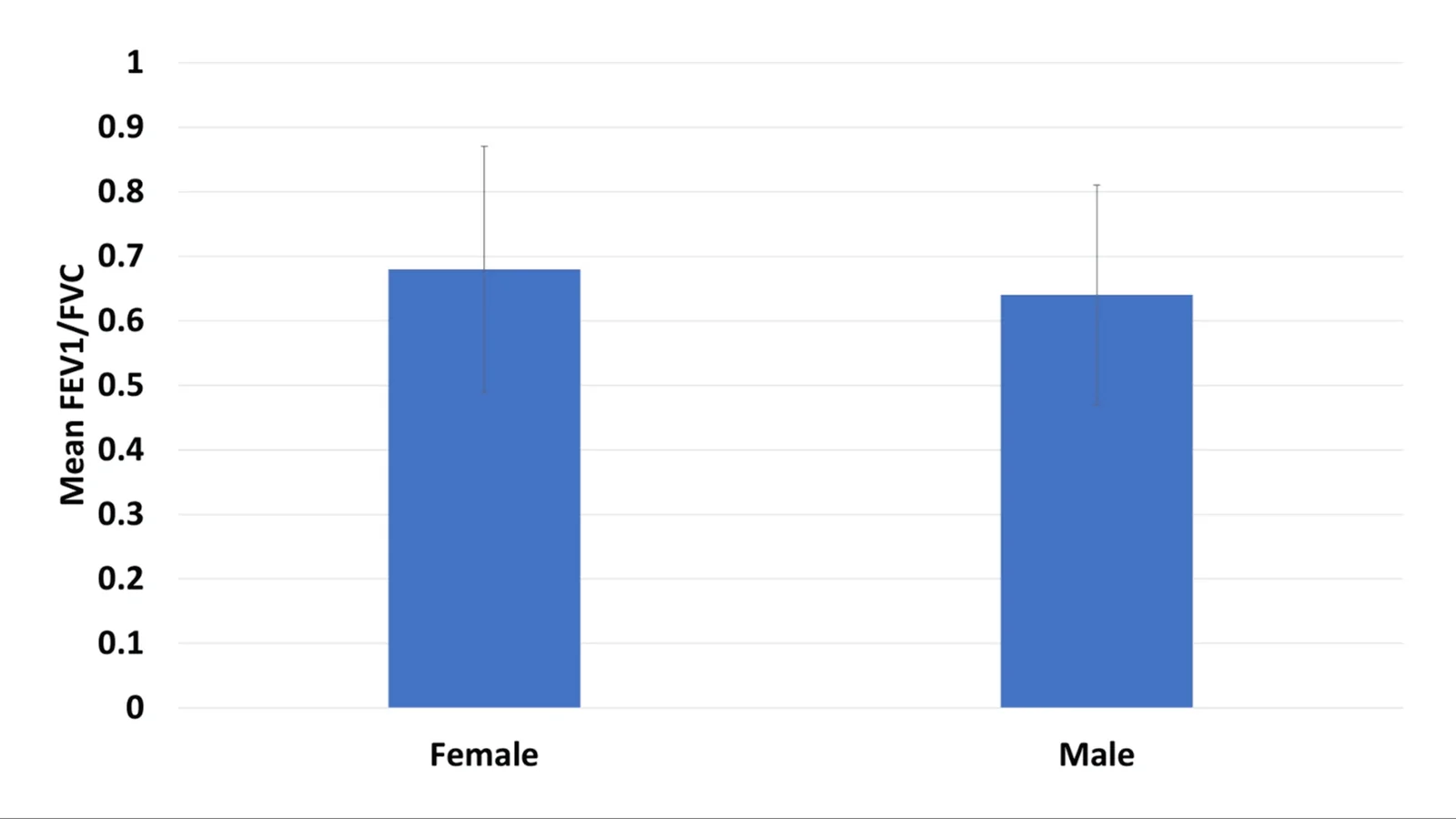
Bar chart representing FEV1/FVC among the patients FEV1: forced expiratory volume in 1 second; FVC: forced vital capacity. N=79. Female n=35 and male n=44. Student's t-test was used. P value=0.32, t=0.98.

The prevalence of ACO across various demographic and clinical factors is tabulated in Table 2. Among the study subjects, previously diagnosed asthmatics were 12 in males and 20 in females, while the figures rose to 30 males and 17 females for previously diagnosed COPD patients. Smoking history was seen in 11 asthmatics and 27 COPD patients, while biomass exposure was seen in 28 asthmatic subjects and 37 COPD subjects. Regarding pulmonary function, 13 asthmatics and 11 COPD patients demonstrated post-bronchodilator reversibility, contrasting with 19 asthmatics and 36 COPD patients who showed irreversibility. The prevalence of ACO was noted to be seven in males and 12 in females with asthma, compared to eight in males and three in females with COPD. The overall prevalence of ACO was 30 for 79 subjects which accounts for 37.97%.

**Table 2:** Prevalence of Asthma-COPD Overlap in the study subjects (Total N=79) N: frequency, COPD: chronic obstructive pulmonary disease, ACO: asthma COPD overlap, FEV1: post-bronchodilator forced expiratory volume in 1 second, FVC: forced vital capacity.

Variable	Previously diagnosed Asthmatic (N=32)	Previously diagnosed COPD (N=47)
Male (N)	12	30
Female (N)	20	17
Positive history of smoking (N)	11	27
Positive history of Biomass exposure (N)	28	37
FEV_1_ Post Bronchodilator Reversibility (N)	13	11
FEV_1_ Post Bronchodilator Irreversibility (N)	19	36
ACO Prevalence in Males (N)	7	8
ACO Prevalence in Females (N)	12	3

## Discussion

Asthma and COPD are respiratory conditions that have distinct clinical characteristics, yet they have a common trait of restricted airflow. Patients who have both COPD and asthma may potentially be diagnosed with ACO, which occurs when the clinical and functional characteristics of both illnesses coincide. ACO was included in many national and international guidelines beginning in 2000 [4].

Cross-sectional research was undertaken to determine the prevalence of ACO among patients with COPD and asthma at the Government District Hospital, Chittoor, and Apollo Medical College. The study spanned a length of one year and included 79 individuals.

The present study examines the sex distribution in a sample population of 79 individuals. The results indicate that 44.30% (35 individuals) are female, while 55.70% (44 individuals) are male, accounting for a total of 100.00% for the dataset. According to the statistics, there are 35 females. Among them, 57.14% (20 individuals) are between the ages of 45 and 55, while 28.57% (10 individuals) are in the 55-65 age group. The age range of 65-75 comprises 8.57% (three persons), whereas the age group over 75 represents 5.71% (two individuals) of the female population. Out of the 44 males, both the age categories of 45-55 and 55-65 each make up 45.45% (20 people), while the age groups of 65-75 and >75 each consist of 4.55% (two individuals).

Among the 35 females included in the present study, 45.71% (16 individuals) are engaged in farming, 22.86% (eight individuals) work in offices, and 31.43% (11 individuals) are categorized as other laborers. Out of the 44 men, 54.55% (24 individuals) work as farmers, 18.18% (eight individuals) work in offices, and 27.27% (12 individuals) are classified as other laborers.

Sevimli et al. (2019) conducted research that yielded similar findings. The ACO group consisted of 27 (67.5%) male and 13 (32.5%) female patients, with an average age of 63.78±10.23 years. The asthma cohort consisted of 64 (21.5%) male and 234 (78.5%) female patients, with an average age of 53.86±13.04 years [8].

In the present study, the distribution of smoking status, classified by sex, was examined in a dataset consisting of 79 individuals. Among the 35 females in the dataset, all of them (100.00%) were non-smokers, with none of them labeled as smokers. Out of the 44 males, 9.09% (four individuals) do not smoke, while 90.91% (40 individuals) are smokers.

In research conducted by Sevimli et al. in 2019, the smoking history was analyzed. The average number of smoking pack-years in the ACO group was 25.83±14.24, with a median value of 22.5 (ranging from 0 to 50) pack-years. On the other hand, the asthma group had an average of 6.44±13.87 pack-years, with a median value of 0 (ranging from 0 to 100) pack-years [8].

In the present study, the distribution of patients previously diagnosed with asthma is examined. The dataset consists of a total of 79 individuals, and they are categorized by sex. Among the 35 females in the dataset, 42.86% (15 individuals) do not have a prior asthma diagnosis, while 57.14% (20 individuals) have been diagnosed with asthma. Out of the 44 males, 72.73% (32 individuals) have not been diagnosed with asthma, whereas 27.27% (12 individuals) have received an asthma diagnosis. 

The present study examines the distribution of biomass exposure over a period of 15 years or more, classified by gender, within a dataset comprising a total of 79 individuals. Out of the 35 females surveyed, 5.71% (two individuals) had not been exposed to biomass for more than 15 years, whereas 94.29% (33 individuals) had experienced this type of exposure. Out of the 44 males, 29.55% (13 people) had not had any contact with biomass for over 15 years, whereas the remaining 70.45% (31 individuals) had been exposed to it.

The present study examines the distribution of patients who have previously been diagnosed with COPD, classified by gender, in a dataset consisting of a total of 79 persons. Out of the 35 females, 51.43% (18 individuals) do not have a previous diagnosis of COPD, whereas 48.57% (17 individuals) have been diagnosed with COPD. Out of the 44 males, 31.82% (14 individuals) have not been previously diagnosed with COPD, whereas 68.18% (30 individuals) have received a COPD diagnosis.

In this study, the average FEV1/FVC ratio for female patients is 0.68, with a standard deviation of 0.19. The average FEV1/FVC ratio for males is 0.64, with a standard deviation of 0.17.

Evaluating spirometric factors is a crucial part of diagnosing ACO. Upon reviewing the literature, it was found that when comparing spirometric variables of patients with asthma, COPD, and ACO, the values of patients with ACO were lower than those of patients with asthma and higher than those of patients with COPD [9].

Small airways are characterized as air passages with a diameter of less than 2 millimeters. Patients with asthma exhibit both major and small airway dysfunctions caused by inflammation. The assessment of small airway dysfunction is done using forced expiratory flow between 25% and 75% of forced vital capacity (FEF₂₅₋₇₅%). The evaluation in the present study of the initial and final FEF₂₅₋₇₅% measurements of patients indicated that individuals with ACO had lower values. The dysfunctions were attributed to exposures, such as smoking and biomass, and were more pronounced in individuals with ACO.

A study conducted by Takeda et al. in 2010 proposed that there is a correlation between uncontrolled asthma and a smaller airway dysfunction. The study also indicated that individuals with this condition experience greater symptoms and have a higher frequency of episodes [10].

In this study, the distribution of COPD reversibility based on sex was examined in a dataset consisting of 47 individuals, including 17 females and 30 men. Among the females, 82.35% (14 persons) had irreversible COPD, whereas 17.65% (three individuals) had reversible COPD. Out of the male population, 73.33% (22 persons) suffer from irreversible COPD, whereas 26.67% (eight individuals) have reversible COPD.

Multiple studies have indicated that individuals diagnosed with ACO experience greater frequencies of episodes and hospitalizations compared to those diagnosed with asthma or COPD [11].

In the PUMA study, a comparison was made between patients with asthma, COPD, and ACO in terms of the frequency of asthma attacks and hospital admissions. The findings revealed that the incidence of patients who had experienced an attack in the preceding year was greater in ACO than in asthma and COPD alone. ACO exhibited a greater frequency of hospital admissions, both for any cause and specifically for illness exacerbation, compared to asthma and COPD alone [12].

In this work, we analyzed the distribution of absolute eosinophilic levels based on sex in a sample consisting of 79 people. Out of the 35 females, 17.14% (six individuals) have an eosinophilic count below 100, 45.71% (16 individuals) have a count between 100 and 200, and 37.14% (13 individuals) have levels higher than 300. Out of the 44 males, 18.18% (eight people) have an eosinophilic count below 100, 34.09% (15 individuals) have counts between 100 and 200, and 47.73% (21 individuals) have counts over 300.

The present study examines the distribution of chest X-ray interpretations based on gender in a sample consisting of 79 people. Out of the 35 females, 48.57% (17 individuals) have COPD alterations in the X-ray, whereas 51.43% (18 individuals) had a normal interpretation. Out of the 44 males, 68.18% (30 people) show signs of COPD on the X-ray, whereas 31.82% (14 individuals) had a normal interpretation.

The prevalence of eosinophilic inflammation is higher in individuals diagnosed with ACO compared to those with COPD. In the management of these patients, inhaled corticosteroids (ICS) play a crucial role. The objective of treating people with ACO is to mitigate both symptoms and the frequency of episodes. Upon reviewing the first treatments of the patients, it was discovered that a majority of patients with ACO were administered high-dose ICS, whereas those with asthma were started on low-to-mild dose ICS. Research has demonstrated that those diagnosed with ACO experience more noticeable symptoms when comparing their respiratory symptoms to those with asthma and COPD. Nevertheless, this results in the administration of elevated medicine dosages in order to manage the illness. With a separate investigation, there was no notable disparity observed among elderly patients with asthma, COPD, and ACO with regards to the utilization of ICS and the daily dosage needed [13,14].

In the present investigation on the prevalence of ACO among different demographic and clinical characteristics, it was shown that among individuals previously diagnosed with asthma, seven men and 12 females showed signs of ACO. However, among those previously diagnosed with COPD, the numbers were eight males and three females. The smoking history data indicates that there are 11 individuals with asthma and 27 individuals with COPD. On the other hand, the biomass exposure data shows a greater prevalence, with 28 individuals with asthma and 37 individuals with COPD. In terms of pulmonary function, there are 13 individuals with asthma and 11 individuals with COPD who exhibit reversibility after using a bronchodilator. In contrast, there are 19 individuals with asthma and 36 individuals with COPD who do not show reversibility. The prevalence of ACO is seen in 30 subjects out of 79 subjects which accounted for 37.97%.

In a previous study conducted by Kiljander et al. in 2015, it was shown that being 60 years old and having smoked more than 20 pack-years were the specific values that could accurately predict the diagnosis of ACO when considering demographic factors. The study found that individuals who were 60 years old had a sensitivity of 63.5% and a specificity of 59.4% in predicting the diagnosis of ACO. The study discovered that the sensitivity for 20 smoking pack-years was 80.8% and the specificity was 42.8%. In the current investigation, a cut-off value of 14 smoking pack-years resulted in a sensitivity of 82.5% and a specificity of 83.6% [15].

The study found that the prevalence of ACO among individuals with asthma was 11.8% [8]. The Gene-Environment Interactions in Respiratory Diseases research, which included 8360 patients, revealed that the rate of overlap between doctor-diagnosed asthma and COPD was 1.6% for those aged 20-44 years, 2.1% for those aged 45-64 years, and 4.5% for those aged 65-84 years. The study found that the prevalence of ACO among patients with asthma was 16% among those aged 20-44 years, 30% among those aged 45-64 years, and 61% among individuals aged 65-84 years [16].

In a multicenter research conducted by Menezes et al. in 2014, across Latin American nations as part of the Proyecto Latinoamericano de Investigation in Obstruccion Pulmonar, a total of 767 patients diagnosed with asthma were categorized into three groups: asthma, COPD, and ACO, with proportions estimated at 1.7%, 12%, and 1.8%, respectively [5].

The PUMA study (Montes de Oca et al., 2017) found that the prevalence of ACO in the general population at risk of COPD in Latin America was 5.3% among individuals with a prior asthma diagnosis and 2.3% among individuals newly diagnosed during the study [12].

In a prior study conducted by Wurst et al. in 2016, the prevalence of ACO was shown to range from 12.1% to 55.2% in patients with COPD and from 13.3% to 61% in patients with asthma [17].

In epidemiological studies, the cause for the varied prevalence of ACO is accounted for by the various diagnostic criteria that researchers employ, as well as the characteristics of the population that is being investigated. For instance, distinct ratios for the frequency of ACO are published for the general population, whereas alternative ratios are offered for individuals who suffer from either asthma or COPD.

COPD and asthma are the two obstructive lung illnesses that are occurring the most often. Although asthma and COPD are typically considered to be two separate diseases, there is a large amount of overlap between the two conditions.

Uncertainty exists over the definition of ACO syndrome, often known as ACOS. Either the diagnosis of COPD in a patient who has already been diagnosed with asthma, or the presence of incompletely reversible airway obstruction accompanied with symptoms or signs of enhanced reversibility of the obstruction, are the two most prevalent definitions of this condition. ACOS is a syndrome that is characterized by chronic airflow limitation with many symptoms that are often associated with asthma and other aspects that are typically associated with COPD. A recent update of the GINA report advised a stepwise approach to the diagnosis of ACOS, and also described ACOS as a syndrome [18].

ACOS is linked with a worse quality of life in terms of health, more frequent exacerbations, greater hospitalization, and higher expenses connected with health care as compared to asthma or COPD alone. Despite the fact that ACOS appears to be clinically extremely relevant, there is a lack of knowledge on the treatment of these patients since they are often excluded from drug trials for asthma or COPD at the present time [19].

The incidence of ACOS is only hinted at by a few pieces of evidence. Hardin et al. discovered that 13% of the COPD patients who participated in the COPD gene research had a history of asthma that had been diagnosed by a physician [20].

In a similar vein, Miravitlles et al. observed that 17.4% of COPD patients who participated in the EPI-SCAN trial disclosed that they had previously been diagnosed with asthma [11].

The frequency of overlap syndrome has been reported to rise with age, which may be a reflection of the fact that asthmatics may acquire fixed airway blockage throughout the course of their lifetimes, particularly if they do not use anti-inflammatory medicine or if they smoke. It was discovered by Soriano and colleagues that as many as 50% of individuals who were diagnosed with obstructive lung disease and were at least 50 years old had more than one obstructive disorder [21].

The fact that asthma and airway hyperresponsiveness are thought to be risk factors for developing COPD may help to explain the high incidence of ACOS. The research conducted by Lange and colleagues has demonstrated that the reduction in pulmonary function is more rapid in asthmatics compared to those who do not have asthma, and that the drop is most rapid among asthmatics who smoke [22].

Sixteen percent of patients with asthma had irreversible airway blockage throughout the course of a 26-year follow-up period, according to the research conducted by Vonk and colleagues. A significant risk factor for the development of COPD is airway hyperresponsiveness, even in the absence of asthma [23].

The most significant risk factor for COPD is smoking. As a result, it should not come as a surprise that the patients with asthma and COPD in our research had smoked more than those with asthma alone. Additional findings from the research conducted by Lee et al. revealed that a higher level of cigarette smoking was associated with the development of fixed airway obstruction in asthmatic patients [24,25].

ACO is a lung disorder that, when present, makes it difficult to breathe. It has characteristics of both asthma and COPD. The majority of the time, being at risk for both asthma and COPD is the cause of this condition. When a person has ACO, the airways, which are the branching tubes that deliver air through the lungs, might get clogged with mucus and constrict. Additionally, the air sacs are prone to being damaged. People report feeling weary and out of breath as a consequence of this. The patient with asthma and COPD has a higher risk of exacerbations than patients with asthma or COPD alone. This is the primary difference in symptoms that distinguishes COPD and asthma. The patient needs to steer clear of risk factors that might result in the development of COPD or exacerbations of the condition on their own.

To put it simply, quitting smoking is the single most essential thing that people can do for their health. If a person already has other underlying risk factors for asthma, smoking can contribute to the development of ACO and make their symptoms more severe. Patients may require assistance from clinicians in order to quit smoking.

Reducing the patient's risk of infection is something that should be done because some illnesses can make ACO symptoms worse. Patients can lower their risk by obtaining immunizations that are appropriate to their condition. The yearly flu shot, the pneumococcal vaccine, and successive doses of the COVID-19 vaccine are some of the vaccinations that are included in this category. Factors that might make a patient's breathing more difficult, such as dust or smog, should be avoided.

Limitations

In the current study, there are certain shortcomings. To begin with, the number of participants was relatively low, and being a single-center study limited our ability to fully evaluate the differences between patients with asthma alone and those with ACOS alone. On the other hand, we were able to confirm that the most prevalent characteristics identified in previous research are also the most common predictors of ACOS. These predictors include older age and a history of heavier smoking. Secondly, there is a possibility that selection bias occurred since we did not analyze consecutive patients.

## Conclusions

In individuals with asthma, the prevalence of ACO is found to be seven in men and 12 in females, but in individuals with COPD, the prevalence is found to be eight in males and three in females. The prevalence of ACO in the present study was 30 for 79 individuals, which accounts for 37.97%. The features of patients with ACO, such as age, gender, smoking status, biomass exposure, and absolute eosinophil count differed considerably among the various source populations. This suggests that ACO, as described in our research, most likely consists of a diverse collection of subpopulations.

ACO, or the underlying continuum of respiratory disease that it reflects, should be the primary focus of research in order to achieve precision in its definition. Additionally, key exposures that are frequent in low- and middle-income countries (LMICs), such as the use of biomass fuel, should be taken into consideration for diagnostic criteria.
